# Will 10 Million People Die a Year due to Antimicrobial Resistance by 2050?

**DOI:** 10.1371/journal.pmed.1002184

**Published:** 2016-11-29

**Authors:** Marlieke E. A. de Kraker, Andrew J. Stewardson, Stephan Harbarth

**Affiliations:** 1 Infection Control Program, Geneva University Hospitals and Faculty of Medicine, Geneva, Switzerland; 2 Infectious Diseases Department, Austin Health, Heidelberg, Australia

## Abstract

Marlieke de Kraker and colleagues reflect on the need for better global estimates for the burden of antimicrobial resistance.

**Summary Points**

- A recent high profile report estimates that, by 2050, 10 million people will die every year due to antimicrobial resistance (AMR) unless a global response to the problem of AMR is mounted.
- There is undoubtedly a large clinical and public health burden associated with AMR, but it is challenging to quantify the associated excess morbidity and mortality.
- When estimates of the burden of AMR are provided, they should be accompanied by clear acknowledgment of the associated uncertainties regarding the incidence of infections, the prevalence of resistance, and the attributable mortality.
- Predictions always require assumptions, but modeling future scenarios using unreliable contemporary estimates is of questionable utility.
- Current global estimates of the burden of AMR are not very informative; we need detailed, reliable data to be able to improve AMR control measures, preferably based on comprehensive, population-based surveillance data from low-, middle-, and high-income countries.

In 2014, Lord Jim O’Neill and his team published a review commissioned by the United Kingdom government entitled, “Antimicrobial Resistance: Tackling a crisis for the health and wealth of nations” (the AMR Review) [1]. The review estimated that antimicrobial resistance (AMR) could cause 10 million deaths a year by 2050. This estimate has become a familiar refrain; it has been quoted repeatedly by lay media, experts, and public health agencies. Frequently, only this specific, frightening conclusion is reproduced from the report, unaccompanied by caveats or confidence intervals. We acknowledge that there is a large clinical and public health burden associated with AMR, that this burden is likely to increase over time, and that urgent action is required [2,3]. However, we contend that unreliable global estimates like those provided in the AMR Review [1] potentially undermine, rather than support, the fight against a post-antibiotic era. In this essay, we will scrutinize the estimations of the burden of AMR provided by the AMR Review [1] and highlight the uncertainties behind these estimates. These uncertainties need to be addressed in order to produce more reliable, detailed, and actionable results.

The first international AMR burden estimates stem from the European Centre for Disease Prevention and Control (ECDC) report “The bacterial challenge: Time to react” [4] in 2009. Because the model estimates reported in the AMR Review [1] are partly based on the ECDC [4] methodology, we will discuss both reports, which estimated the burden of AMR in Europe and the world, respectively. The AMR Review [1] includes estimates produced by two different consultancy firms: RAND and KPMG. Because the most quoted phrase is derived from the KPMG estimates [5], the KPMG model will be represented whenever we refer to the AMR Review [1] throughout the remainder of this essay.

## The Number of Bloodstream Infections in the World

**Fig 1:**
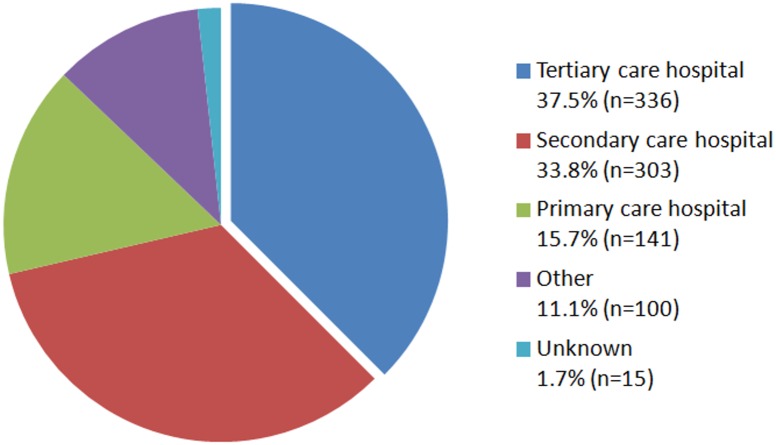
The distribution of hospital care level among hospitals reporting antimicrobial susceptibility and denominator data (subset of all hospitals) to EARS-Net (2013/2014) [6].

Despite being published five years apart, the ECDC report [4] and AMR Review [1] are based on very similar assumptions. First, for both reports, data about the number of infections by selected bacterial pathogens (*Escherichia coli*, *Klebsiella pneumoniae*, and *Staphylococcus aureus*) for European countries are derived from the European Antimicrobial Resistance Surveillance network (EARS-Net). However, this is not a population-based surveillance network; EARS-Net only records invasive infections diagnosed in hospitals and for a variable proportion of the total number of hospitals in each country. Tertiary care hospitals are much more likely to participate in this program than smaller hospitals (Fig 1). In order to generate national estimates for number of infections, both reports extrapolate these EARS-Net estimates through use of the reported catchment population of EARS-Net. This means that the incidence rate of infections in predominantly tertiary care hospitals is applied to the whole country. Moreover, overlapping catchment areas between hospitals and uncertainty in catchment population estimates are ignored. For non-EARS-Net countries, included in the global perspective of the AMR Review [1], an even more crude approach was used; the number of infections was based on the EARS-Net average infection rates per 100,000 people multiplied by population size.

## The Number of Resistant Bloodstream Infections: Resistance Proportions Versus Incidence Rates

The second step to determine the burden of AMR is to estimate the number of infections caused by pathogens that are resistant to the antibiotic (or antibiotic class) of interest. For the AMR Review [1], third-generation cephalosporin-resistant *E*. *coli* and *K*. *pneumoniae* and methicillin-resistant *S*. *aureus* (MRSA) were selected. The models in both the ECDC report and AMR Review multiplied the national number of bloodstream infections (BSIs) by the national resistance proportions as reported by EARS-Net and the WHO, for European and non-European countries, respectively.

A general problem with utilizing resistance proportion data from AMR surveillance networks is variability in the frequency of blood culture sampling. Blood culturing habits can greatly influence the estimated proportion of resistance. Consider the two following scenarios. If few patients are cultured—for example, the very ill or those failing standard empiric therapy—resistant strains will be over-represented, resulting in a high resistance proportion. At the other extreme, if all patients are cultured, this will dilute the number of resistant pathogens identified, and the estimated resistance proportion will be lower. In the same scenarios, resistance rates (i.e., number of resistant isolates/1,000 patient-days) will still be correct (Table 1). Therefore, aggregated AMR estimates at, for example, the national level are much more reliable when they are based on incidence rates. Unfortunately, this kind of data is seldom available, often resulting in overestimates of the resistance problem, especially in resource-poor settings.

**Table 1:** Influence of culture rate on resistance proportions and resistance rates.

Culture rate	Proportion: Resistant isolates/total isolates	Incidence: Resistant isolates/patient-days
**High**	Too low	“True”
**Adequate**	“True”	“True”
**Low (still detecting all resistant isolates)**	Too high	“True”
**Very low (not detecting all resistant isolates)**	Very high	Too low

## Extrapolation from Bloodstream Infections to Infections at Other Sites

Like most laboratory-based infection surveillance networks for health care–associated infections, EARS-Net [6] only includes BSIs, because these are most easily defined and more likely to have substantial clinical impact. However, these infections only constitute the tip of the iceberg. To overcome this problem in the AMR Review [1], the number of resistant lower respiratory tract infections (LRTIs), surgical site infections (SSIs), and urinary tract infections (UTIs) was calculated by applying a ratio between each one and the estimated, national numbers of resistant BSIs. The AMR Review refers to the ECDC report [4] as methodological background without further comment. For third-generation cephalosporin-resistant *E*. *coli* and *K*. *pneumoniae*, and MRSA, the ECDC based these ratios on data from two studies: a multicenter study in Brooklyn (including 12 extended spectrum betalactamase-positive *K*. *pneumonia* BSIs) [7] and a single-center study in Spain (including four MRSA BSIs) [8], with data from 1999 and 2002, respectively.

## Attributable Mortality

Using the three problematic calculation steps discussed above, the (inter)national number of resistant BSIs, LRTIs, SSIs, and UTIs was estimated for 2007 (ECDC) and 2012 (AMR Review), respectively. The next step in estimating the burden of AMR is to multiply estimates of the absolute number of resistant infections by a certain “per-infection” burden measure. The AMR Review [1] and ECDC [4] report both focused on mortality, utilizing published mortality data. However, most scientific research articles, and specifically the ones referred to by ECDC, only report crude mortality proportions or adjusted odds ratios (ORs) for patients with resistant infections versus those with susceptible infections. These ORs cannot be easily transformed to attributable mortality proportions [9]. Moreover, these ORs express the attributable mortality due to a specific antimicrobial resistance profile of a specific pathogen, and this effect is a mix of timing of appropriate therapy and host- and pathogen-related factors, with low external validity. The ECDC report [4] utilized data from different studies to generate infection- and pathogen-specific attributable mortality rates, ranging from 0.2% to 30%. The methodology for this process was not reported. Nevertheless, the same attributable mortality estimates were utilized in the AMR Review [1]. In addition to the debatable external validity of these attributable mortality estimates, the internal validity is questionable, as none of the included studies took into account methodological challenges such as time-dependent bias or competing outcomes [10]. Time-dependent bias occurs when people have to survive for a certain amount of time to be able to be exposed, such as time from hospital admission to health care–associated infection. If this “immortal time” is ignored, the impact of infection will be underestimated. A competing event for hospital mortality is discharge alive; patients who are discharged alive will have zero risk of hospital mortality. If this fact is ignored in the analyses, mortality may be overestimated.

## Future Scenarios

Although the ECDC report [4] only provided contemporary estimates, the AMR Review [1] also presented future scenarios; based on already uncertain burden estimates, another layer of assumptions was added to make forecasts decades into the future about rise of the number of infections and associated deaths. The AMR Review considered four different scenarios: an absolute rise in resistance levels of 40% for all species under study or 100% resistance, with both of these scenarios combined with either stable or doubled infection rates. To date, there is no empirical data supporting any of these scenarios. Furthermore, each scenario assumes that the mortality risk per infection will remain unchanged, despite evidence that mortality rates associated with BSIs and sepsis are decreasing due to improved supportive care [11,12]. This trend might be even more marked in middle-income countries due to improvements in public health and health care systems. The scenario that seems to be underlying the most often quoted line entails a sharp initial rise of current resistance rates by 40 percentage points, after which rates remain stable until 2050, and doubled infection rates.

## Scientific Scrutiny

Although the results from these burden reports are often cited, none of these burden estimates themselves have been published in peer-reviewed literature. The question is whether they would endure scientific scrutiny. KPMG [5] states that external experts were invited to provide input for the applied models, but there is no statement about independent peer review of the final results. In the AMR Review [1] it is acknowledged that the reported numbers are “broad brush estimates,” that “more detailed and robust work will no doubt be done by academic researchers,” and that there is a lack of data, urging for improvement of infection surveillance. Nevertheless, it is not clearly reported how existing uncertainties in each of the applied steps could affect their estimates. In the chapter presenting the “10 million” estimate, exact numbers are reported without clear reference to background data, assumptions, or scenarios. Confidence intervals or results from sensitivity analyses are likewise omitted. In our opinion, burden estimates for such an important, “hot” topic should undergo scrutiny by independent experts before being made publicly available. The scientific community would have requested clearer communication of uncertainties in their assumptions and, more importantly, uncertainties about their baseline infection rates and attributable mortality estimates. No doubt this would have resulted in substantial confidence intervals for the “10 million” estimate.

## The Way Forward

Clearly, there is a need for more reliable AMR burden estimates, including uncertainty boundaries, and more careful modeling of future scenarios, including sensitivity analyses. The key prerequisite for that is more comprehensive antimicrobial resistance surveillance data, especially for low- and middle-income countries and especially for community-acquired infections. The next step would be to provide AMR-related morbidity and mortality data through these population-based surveillance networks. Demographic data would further enable age- and gender-specific estimates, making detailed results available upon which the most effective AMR control measures can be built. Until these types of data are available, global AMR burden estimates are not reliable and will not be able to inform meaningful action. At a minimum, these estimates should be reported with more transparency and be interpreted with caution.

In September 2016, the UN convened a General Assembly in New York to summon strong political commitment in addressing AMR. Although it is too early to evaluate any true advancement, countries reaffirmed their commitment to develop national action plans on AMR based on the WHO Global Action Plan on Antimicrobial Resistance (2015). This plan has five objectives, of which the first two focus on awareness and understanding of AMR through surveillance and research. At the same time, a group of (inter)national organizations have joined forces to form the Conscience of Antimicrobial Resistance Action (CARA) to hold the UN and other governmental bodies to the commitments they have made. Hopefully, these renewed national commitments and the positive influence from CARA will result in invigorated initiatives for accurate, population-based AMR surveillance, including burden of disease monitoring on a global scale.

## Abbreviations

AMR: antimicrobial resistance
BSI: bloodstream infection
CARA: Conscience of Antimicrobial Resistance Action
EARS-Net: European Antimicrobial Resistance Surveillance Network
ECDC: European Centre for Disease Prevention and Control
LRTI: lower respiratory tract infection
MRSA: methicillin-resistant *Staphylococcus aureus*
OR: odds ratio
SSI: surgical site infection
UTI: urinary tract infection

## References

[1] O'Neill J. Review on Antimicrobial Resistance Antimicrobial Resistance: Tackling a crisis for the health and wealth of nations. London: Review on Antimicrobial Resistance 2014 Available from: https://amr-review.org/sites/default/files/AMR%20Review%20Paper%20-%20Tackling%20a%20crisis%20for%20the%20health%20and%20wealth%20of%20nations_1.pdf

[2] De Kraker MEA, Davey PG, Grundmann H. Mortality and Hospital Stay Associated with Resistant Staphylococcus aureus and Escherichia coli Bacteremia: Estimating the Burden of Antibiotic Resistance in Europe. PLoS Med. 2011; 8: e1001104 10.1371/journal.pmed.1001104 22022233 PMC3191157

[3] Stewardson AJ, Allignol A, Beyersmann J, Graves N, Schumacher M, et al The health and economic burden of bloodstream infections caused by antimicrobial-susceptible and non-susceptible Enterobacteriaceae and Staphylococcus aureus in European hospitals, 2010 and 2011: a multicentre retrospective cohort study. Eurosurveillance. 2016; 21: pii = 30319. 10.2807/1560-7917.ES.2016.21.33.30319 PMC4998424 27562950

[4] European Centre for Disease Prevention and Control (ECDC) and European Medicines Agency (EMEA) Joint Working Group. ECDC/ EMEA Joint Technical Report: The bacterial challenge: Time to react. Stockholm: ECDC. 2009. Available from: http://ecdc.europa.eu/en/publications/Publications/0909_TER_The_Bacterial_Challenge_Time_to_React.pdf

[5] KPMG LLP. The global economic impact of anti-microbial resistance. London: KPMG LLP UK, research commissioned by the Wellcome Trust, as part of an independent review into anti-microbial resistance supported by the Department of Health and the Wellcome Trust. 2014. Available from: https://home.kpmg.com/content/dam/kpmg/pdf/2014/12/amr-report-final.pdf

[6] European Centre for Disease Prevention and Control (ECDC) Antimicrobial Resistance Surveillance in Europe 2014. Annual report of the European Antimicrobial Resistance Surveillance Network (EARS-Net). Stockholm: ECDC. 2015. Available from: http://ecdc.europa.eu/en/publications/publications/antimicrobial-resistance-europe-2014.pdf

[7] The Brooklyn Antibiotic Task Force. The cost of antibiotic resistance: effect of resistance among Staphylococcus aureus, Klebsiella pneumoniae, Acinetobacter baumannii, and Pseudmonas aeruginosa on length of hospital stay. Infect Control Hosp Epidemiol. 2002; 23: 106–108. 10.1086/502018 11893146

[8] Gavalda L, Masuet C, Beltran J, Garcia M, Garcia D, et al Comparative cost of selective screening to prevent transmission of methicillin-resistant Staphylococcus aureus (MRSA), compared with the attributable costs of MRSA infection. Infect Control Hosp Epidemiol. 2006; 27: 1264–1266. 10.1086/507968 17080390

[9] Samore M, Harbarth S. A methodologically focused review of the literature in hospital epidemiology and infection control Hospital Epidemiology and Infection Control by Mayhall CG. Philadelphia, PA: Lippincott Williams & Wilkins; 2004 pp. 1659–1702.

[10] Schumacher M, Allignol A, Beyersmann J, Binder N, Wolkewitz M. Hospital-acquired infections—appropriate statistical treatment is urgently needed! Int J Epidemiol. 2013; 42: 1502–1508. 10.1093/ije/dyt111 24038717

[11] Gaieski DF, Edwards JM, Kallan MJ, Carr BG. Benchmarking the incidence and mortality of severe sepsis in the United States. Crit Care Med. 2013; 41: 1167–1174. 10.1097/CCM.0b013e31827c09f8 23442987

[12] Kaukonen KM, Bailey M, Suzuki S, Pilcher D, Bellomo R. Mortality related to severe sepsis and septic shock among critically ill patients in Australia and New Zealand, 2000–2012. JAMA. 2014; 311: 1308–1316. 10.1001/jama.2014.2637 24638143

